# Supplementary material for: Mammalian cell display with automated oligo design and library assembly allows for rapid residue level conformational epitope mapping
Source: Commun Biol. 2024 Jul 3;7:805. doi: 10.1038/s42003-024-06508-8 (PMC11222437; doi:10.1038/s42003-024-06508-8)
Supplement: Supplementary file 2 — Supplementary information [file 42003_2024_6508_MOESM2_ESM.pdf]

Mammalian cell display with automated oligo design and library assembly allows for rapid residue level conformational epitope mapping

Supplementary information

Niklas Berndt Thalén[1†], Maximilian Karlander[1†], Magnus Lundqvist[1], Helena Persson[2], Camilla Hofström[2], S. Pauliina Turunen[2], Magdalena Godzwon[3], Anna-Luisa Volk[1], Magdalena Malm[1], Mats Ohlin[3], Johan Rockberg[1#]

[1]KTH - Royal Institute of Technology, Dept. Protein science; Stockholm; SE-106 91; Sweden
[2]Science for Life Laboratory, Drug Discovery and Development Platform & School of Biotechnology, KTH-Royal Institute of Technology, Stockholm, Sweden
[3]Department of Immunotechnology, Lund University, Lund, Sweden

[†] Joint authors

[#] To whom correspondence should be addressed: Prof. Johan Rockberg
KTH Royal Institute of Technology, Dept Protein science, Roslagstullsbacken 21, 10691, Stockholm Sweden
Phone: +46 8 790 99 88

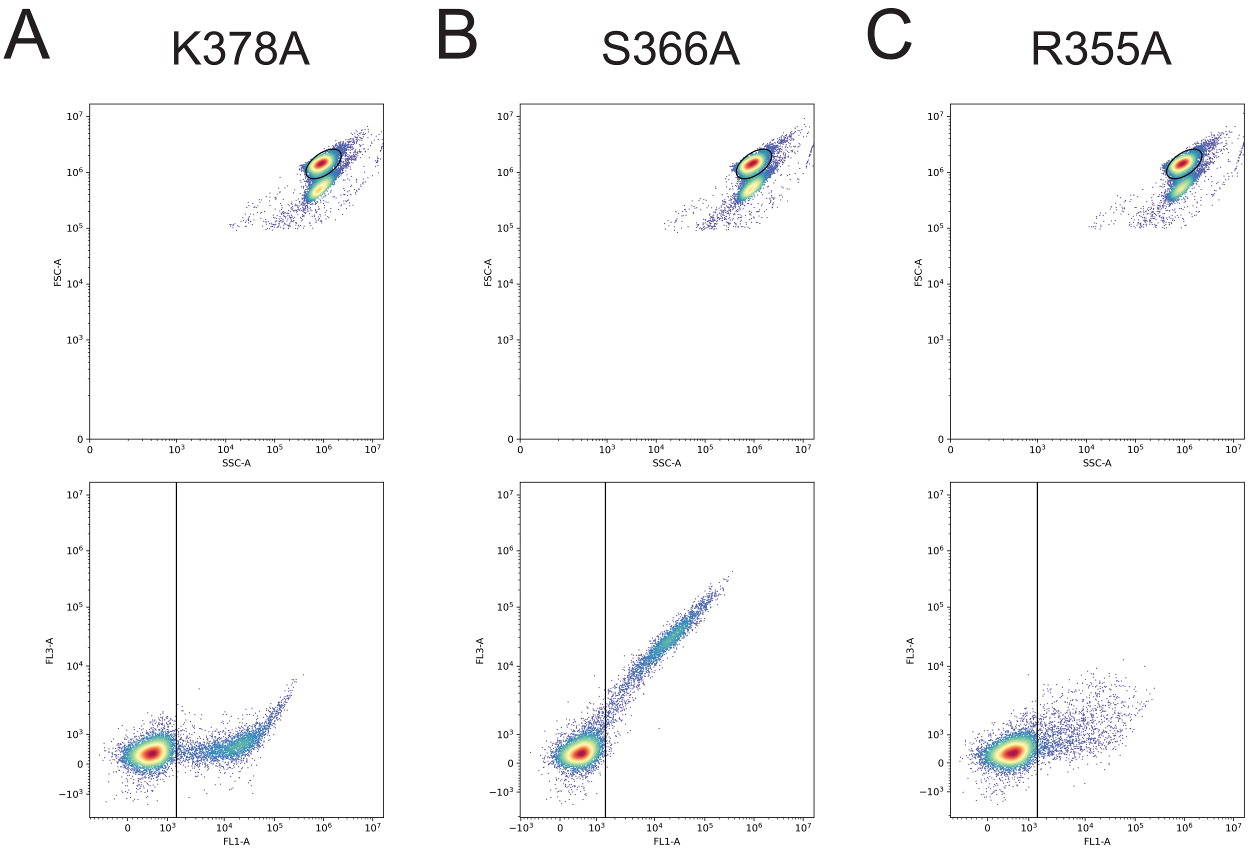

	Events	Percent gated	From previous
No gate	18578	100.0%	100.0%
Eliptical gate (1)	10558	56.83%	56.83%
Linear gate (2)	2842	15.3%	26.92%

	Events	Percent gated	From previous
No gate	18152	100.0%	100.0%
Eliptical gate (1)	10704	58.97%	58.97%
Linear gate (2)	2831	15.6%	26.45%

	Events	Percent gated	From previous
No gate	16395	100.0%	100.0%
Eliptical gate (1)	10572	64.48%	64.48%
Linear gate (2)	1480	9.03%	14.0%

Figure S1: Examples of different residue properties as observed in flow cytometry for CR3022. The top row shows cell population gating based on forward scatter/side scatter. The middle row shows the populations based on the two fluorescent channels. FL1-A refers to Alexa488 coupled to the anti-HA antibody used for determining protein expression. FL3-A refers to Alexa647 coupled to the anti-human antibody binding the tested antibody and is used to determine binding. The lowest rows shows number of events present in the gates. A) Mutation K378A is an example of an expressing, binding-disrupting residue. B) Mutation S366A is an example of an expressing residue not disrupting the binding interaction. C) Mutation R355A is an example of a residue which could not properly be expressed on the cell surface and therefore not be determined as being binding-disruptining or not.

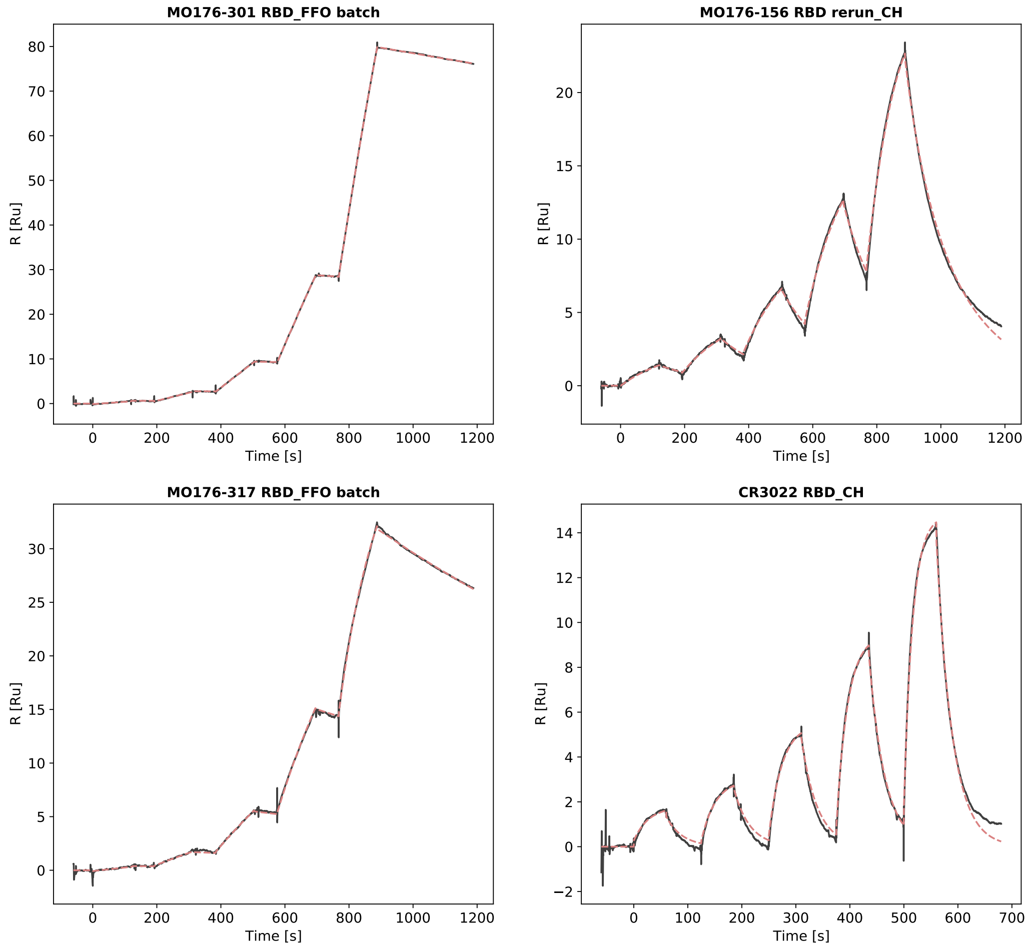

Figure S2: Single cycle kinetics assay for determination of affinity and kinetic constants for all four anti-RBD antibodies. All four antibodies display concentration dependent binding to RBD.
