# Supplementary material for: Mammalian cell display with automated oligo design and library assembly allows for rapid residue level conformational epitope mapping
Source: Commun Biol. 2024 Jul 3;7:805. doi: 10.1038/s42003-024-06508-8 (PMC11222437; doi:10.1038/s42003-024-06508-8)
Supplement: Supplementary file 3 — Description of additional supplementary files [file 42003_2024_6508_MOESM3_ESM.pdf]

Description of Additional Supplementary Files

File name: Supplementary Data 1

Description: Table over all primers used for the alanine scanning mutagenesis including key parameters. As generated by Kozane. Referred to in the manuscript as Supplementary data 1.

File name: Supplementary Data 2

Description: All flow cytometry plots generated for the epitope determination. Referred to in the manuscript as Supplementary data 2.
