# Supplementary material for: Mammalian cell display with automated oligo design and library assembly allows for rapid residue level conformational epitope mapping
Source: Commun Biol. 2024 Jul 3;7:805. doi: 10.1038/s42003-024-06508-8 (PMC11222437; doi:10.1038/s42003-024-06508-8)
Supplement: Supplementary file 5 — Supplementary data 2 [file 42003_2024_6508_MOESM5_ESM.html]

 

 

 

 
 
 


 Mammalian cell display with automated oligo design and library assembly allows for rapid residue level conformational epitope mapping 

 
 
 
 
 
 
 
 
 
 
 
 
 
 

 

 
 


 


 

 

 


 


 

 


 


 
 
 
 
 
 

 


 


 Mammalian cell display with automated oligo
design and library assembly allows for rapid residue level
conformational epitope mapping 
 Supplementary data 2 
  Niklas Berndt
Thalén 1† , Maximilian Karlander 1† , Magnus
Lundqvist 1 , Helena Persson 2 , 
Camilla Hofström 2 , S. Pauliina Turunen 2 , Magdalena
Godzwon 3 , Anna-Luisa Volk 1 , 
Magdalena Malm 1 , Mats Ohlin 3 , Johan
Rockberg 1#  
 
 1 KTH - Royal Institute of Technology, Dept. Protein science;
Stockholm; SE-106 91; Sweden 
 2 Science for Life Laboratory, Drug Discovery and Development
Platform & School of 
Biotechnology, KTH-Royal Institute of Technology, Stockholm,
Sweden 
 3 Department of Immunotechnology, Lund University, Lund,
Sweden 
 
 †  Joint authors 
 
 #  To whom correspondence should be addressed: Prof. Johan
Rockberg 
KTH Royal Institute of Technology, Dept Protein science,
Roslagstullsbacken 21, 10691, 
Stockholm Sweden 
Phone: +46 8 790 99 88  

 


 
  1  Description of
data 
 This document contains all flow cytometry plots generated for this
article. They are organized based on the antibody/ligand and the title
of each plot refers the position tested. 
 
 Expression - Alexa 488 refers to the fluorophore used to determine
expression. A rabbit anti-HA antibody was used as primary antibody and
an Alexa488 conjugated anti-rabbit antibody was sused as secondary
antibody. 
 Binding - Alexa 647 refers to the fluorophore used to determine
binding. As all antibodies used are human and ACE2 was human FC-coupled
an Alexa647 conjugated anti-human antibody was used for detection. 
 
 
 
  2  ACE2 
   
   
   
   
   
   
   
   
   
   
   
   
   
   
   
   
   
   
   
   
   
   
   
   
   
   
   
   
   
   
   
   
   
   
   
   
   
   
   
   
   
   
   
   
   
   
   
   
   
   
   
   
   
   
   
   
   
   
   
   
   
   
   
   
   
   
   
   
   
   
   
   
   
   
   
   
   
   
   
   
   
   
   
   
 
 
  3  CR3022 
   
   
   
   
   
   
   
   
   
   
   
   
   
   
   
   
   
   
   
   
   
   
   
   
   
   
   
   
   
   
   
   
   
   
   
   
   
   
   
   
   
   
   
   
   
   
   
   
   
   
   
   
   
   
   
   
   
   
   
   
   
   
   
   
   
   
   
   
   
   
   
   
   
   
   
   
   
   
   
   
   
   
   
   
 
 
  4  MO176_156 
   
   
   
   
   
   
   
   
   
   
   
   
   
   
   
   
   
   
   
   
   
   
   
   
   
   
   
   
   
   
   
   
   
   
   
   
   
   
   
   
   
   
   
   
   
   
   
   
   
   
   
   
   
   
   
   
   
   
   
   
   
   
   
   
   
   
   
   
   
   
   
   
   
   
   
   
   
   
   
   
   
   
   
   
 
 
  5  MO176_301 
   
   
   
   
   
   
   
   
   
   
   
   
   
   
   
   
   
   
   
   
   
   
   
   
   
   
   
   
   
   
   
   
   
   
   
   
   
   
   
   
   
   
   
   
   
   
   
   
   
   
   
   
   
   
   
   
   
   
   
   
   
   
   
   
   
   
   
   
   
   
   
   
   
   
   
   
   
   
   
   
   
   
   
   
 
 
  6  MO176_317 
   
   
   
   
   
   
   
   
   
   
   
   
   
   
   
   
   
   
   
   
   
   
   
   
   
   
   
   
   
   
   
   
   
   
   
   
   
   
   
   
   
   
   
   
   
   
   
   
   
   
   
   
   
   
   
   
   
   
   
   
   
   
   
   
   
   
   
   
   
   
   
   
   
   
   
   
   
   
   
   
   
   
   
   
 


 
 

 

 

 

 

 

 

 
 

 
 
