# Supplementary material for: Mammalian cell display with automated oligo design and library assembly allows for rapid residue level conformational epitope mapping
Source: Commun Biol. 2024 Jul 3;7:805. doi: 10.1038/s42003-024-06508-8 (PMC11222437; doi:10.1038/s42003-024-06508-8)
Supplement: Supplementary file 6 — Reporting summary [file 42003_2024_6508_MOESM6_ESM.pdf]

nature portfolio

Corresponding author(s): Professor Johan Rockberg

Last updated by author(s): May 21, 2024

Reporting Summary

Nature Portfolio wishes to improve the reproducibility of the work that we publish. This form provides structure for consistency and transparency in reporting. For further information on Nature Portfolio policies, see our Editorial Policies and the Editorial Policy Checklist.

Statistics

For all statistical analyses, confirm that the following items are present in the figure legend, table legend, main text, or Methods section.

n/a	Confirmed	
☐	☒	The exact sample size (n) for each experimental group/condition, given as a discrete number and unit of measurement
☒	☐	A statement on whether measurements were taken from distinct samples or whether the same sample was measured repeatedly
☐	☒	The statistical test(s) used AND whether they are one- or two-sided<br>*Only common tests should be described solely by name; describe more complex techniques in the Methods section.*
☒	☐	A description of all covariates tested
☐	☒	A description of any assumptions or corrections, such as tests of normality and adjustment for multiple comparisons
☐	☒	A full description of the statistical parameters including central tendency (e.g. means) or other basic estimates (e.g. regression coefficient) AND variation (e.g. standard deviation) or associated estimates of uncertainty (e.g. confidence intervals)
☐	☒	For null hypothesis testing, the test statistic (e.g. F, t, r) with confidence intervals, effect sizes, degrees of freedom and P value noted<br>*Give P values as exact values whenever suitable.*
☒	☐	For Bayesian analysis, information on the choice of priors and Markov chain Monte Carlo settings
☒	☐	For hierarchical and complex designs, identification of the appropriate level for tests and full reporting of outcomes
☒	☐	Estimates of effect sizes (e.g. Cohen's d, Pearson's r), indicating how they were calculated

Our web collection on statistics for biologists contains articles on many of the points above.

Software and code

Policy information about availability of computer code

Data collection

CytExpert v.2.4.0.28 (Beckman Coultier, Inc.)
Sierra SPR Control v3.4.7.0 (Bruker)
Biacore T200 Control Software, v.3.2.1 (Cytiva)
Wallac EnVision Manager v1.12 (Perkin Elmer).

Data analysis

Sierra Analyser v3.4.5 (Bruker)
BIAevaluation, v. 3.2.1 (Cytiva)
Excel for Mac v16.40 (Microsoft)
Geneious Prime v.2023.0.1 (Biomatters Ltd)
Kozane (https://www.kozane.app)
PyMOL (open source v.2.5.0)
Python (3.9.7) using the packages:
- Pandas (v.1.5.2)
- MatPlotLib (v.3.6.2)
- Seaborn (v.0.12.1)
- Numpy (v.1.23.5)
- tomlkit (v.0.11.6)
- Scipy (v. 1.9.3)
- FlowCal (v.1.3.0)

- tqdm (v.4.64.1)

For manuscripts utilizing custom algorithms or software that are central to the research but not yet described in published literature, software must be made available to editors and reviewers. We strongly encourage code deposition in a community repository (e.g. GitHub). See the Nature Portfolio guidelines for submitting code & software for further information.

Data

Policy information about availability of data

All manuscripts must include a data availability statement. This statement should provide the following information, where applicable:

- Accession codes, unique identifiers, or web links for publicly available datasets
- A description of any restrictions on data availability
- For clinical datasets or third party data, please ensure that the statement adheres to our policy

The data supporting the findings of the presented study are available within the article and its Supplementary Information files and from the corresponding authors upon reasonable request. Source data are provided with this paper.

Research involving human participants, their data, or biological material

Policy information about studies with human participants or human data. See also policy information about sex, gender (identity/presentation), and sexual orientation and race, ethnicity and racism.

Reporting on sex and gender	N/A
Reporting on race, ethnicity, or other socially relevant groupings	N/A
Population characteristics	N/A
Recruitment	N/A
Ethics oversight	N/A

Note that full information on the approval of the study protocol must also be provided in the manuscript.

Field-specific reporting

Please select the one below that is the best fit for your research. If you are not sure, read the appropriate sections before making your selection.

☒ Life sciences ☐ Behavioural & social sciences ☐ Ecological, evolutionary & environmental sciences

For a reference copy of the document with all sections, see nature.com/documents/nr-reporting-summary-flat.pdf

Life sciences study design

All studies must disclose on these points even when the disclosure is negative.

Sample size	No sample size calculation performed.
Data exclusions	N/A
Replication	N/A
Randomization	N/A
Blinding	N/A

Reporting for specific materials, systems and methods

We require information from authors about some types of materials, experimental systems and methods used in many studies. Here, indicate whether each material, system or method listed is relevant to your study. If you are not sure if a list item applies to your research, read the appropriate section before selecting a response.

Materials & experimental systems

n/a	Involved in the study
☐	☒ Antibodies
☐	☒ Eukaryotic cell lines
☒	☐ Palaeontology and archaeology
☒	☐ Animals and other organisms
☒	☐ Clinical data
☒	☐ Dual use research of concern
☒	☐ Plants

Methods

n/a	Involved in the study
☒	☐ ChIP-seq
☐	☒ Flow cytometry
☒	☐ MRI-based neuroimaging

Antibodies

Antibodies used

Competitive HTRF: S1-binding antibody fragments scFvs, Fabs, human IgG1 CR3022 (srbd-mab1, InVivoGen (Toulouse, France)) and mouse IgG2b CR3022 (Ab01680-3.0, Absolute Antibodies (Wilton, UK))
HTRF detection: europium-conjugated anti-FLAG antibody (Cisbio, 61FG2KLA (Codolet, France)) for scFv and europium-conjugated anti-kappa antibody (Cisbio #61KAPKAA (Codolet, France)) for Fab
Flow cytometry: Rabbit anti-HA antibody (HA88-342, Invitrogen (Carlsbad, CA, USA)) as primary antibody for expression, Alexa anti-rabbit 488 (A32731, Invitrogen (Carlsbad, CA, USA)) as secondary antibody for expression, Alexa anti-human 647 (A21445, Invitrogen (Carlsbad, CA, USA)) as secondary antibody for binding

Validation

All antibodies were validated as stated in vendors website for respective application.

Eukaryotic cell lines

Policy information about cell lines and Sex and Gender in Research

Cell line source(s)

ExpiCHO Cells- Derived from CHO-S (Thermo fischer)

Authentication

Was not authenticated

Mycoplasma contamination

All cell lines mycoplasma negative

Commonly misidentified lines
(See ICLAC register)

N/A

Flow Cytometry

Plots

Confirm that:

☒ The axis labels state the marker and fluorochrome used (e.g. CD4-FITC).

☒ The axis scales are clearly visible. Include numbers along axes only for bottom left plot of group (a 'group' is an analysis of identical markers).

☒ All plots are contour plots with outliers or pseudocolor plots.

☒ A numerical value for number of cells or percentage (with statistics) is provided.

Methodology

Sample preparation

ExpiCho cells were harvested and washed twice in PBS.Primary antibodies were added against target protein and expression tag. The cells were washed twice followed by incubation with secondary antiboides. The cells were washed twice before loading in the flow cytometer.

Instrument

CytoFLEX S, Serial number: BF06033, ID:81937078

Software

Kaluza Analysis 2.1 (Beckman Coulter Life Science, Germany)
Python (3.9.7) using FlowCal (v.1.3.0)

Cell population abundance

No sorting was performed.

Gating strategy

FSC and SSC gating for single cells. Vertical linear gate on FL1-A to remove non-expressing population. Special case eliptic gate was on FL1-A/FL3-A to separate double populations for one antibody tested.

☒ Tick this box to confirm that a figure exemplifying the gating strategy is provided in the Supplementary Information.
